# Hypotaurine evokes a malignant phenotype in glioma through aberrant hypoxic signaling

**DOI:** 10.18632/oncotarget.7710

**Published:** 2016-02-25

**Authors:** Peng Gao, Chunzhang Yang, Cody L. Nesvick, Michael J. Feldman, Saman Sizdahkhani, Huailei Liu, Huiying Chu, Fengxu Yang, Ling Tang, Jing Tian, Shiguang Zhao, Guohui Li, John D. Heiss, Yang Liu, Zhengping Zhuang, Guowang Xu

**Affiliations:** ^1^ Key Laboratory of Separation Science for Analytical Chemistry, Dalian Institute of Chemical Physics, Chinese Academy of Sciences, Dalian, China; ^2^ Surgical Neurology Branch, National Institute of Neurological Disorders and Stroke, National Institutes of Health, Bethesda, MD, USA; ^3^ Department of Neurosurgery, the First Affiliated Hospital of Harbin Medical University, Harbin, China; ^4^ Laboratory of Molecular Modeling and Design, State Key Laboratory of Molecular Reaction Dynamics, Dalian Institute of Chemical Physics, Chinese Academy of Science, Dalian, China; ^5^ School of Bioengineering, Dalian Polytechnic University, Dalian, China; ^6^ Clinical Laboratory, Dalian Sixth People's Hospital, Dalian, China; ^7^ Neuro-Oncology Branch, National Cancer Institute, NIH, Bethesda, MD, USA

**Keywords:** hypoxia, hypoxia-inducible factors, hypotaurine, metabolomics, glioma

## Abstract

Metabolomics has shown significant potential in identifying small molecules specific to tumor phenotypes. In this study we analyzed resected tissue metabolites using capillary electrophoresis-mass spectrometry and found that tissue hypotaurine levels strongly and positively correlated with glioma grade. *In vitro* studies were conducted to show that hypotaurine activates hypoxia signaling through the competitive inhibition of prolyl hydroxylase domain-2. This leads to the activation of hypoxia signaling as well as to the enhancement of glioma cell proliferation and invasion. In contrast, taurine, the oxidation metabolite of hypotaurine, decreased intracellular hypotaurine and resulted in glioma cell growth arrest. Lastly, a glioblastoma xenograft mice model was supplemented with taurine feed and exhibited impaired tumor growth. Taken together, these findings suggest that hypotaurine is an aberrantly produced oncometabolite, mediating tumor molecular pathophysiology and progression. The hypotaurine metabolic pathway may provide a potentially new target for glioblastoma diagnosis and therapy.

## INTRODUCTION

Gliomas are the most common primary brain malignancies in adults [1]. Advances have recently been made in elucidating various glioma phenotypes, including the development of the World Health Organization (WHO) glioma grading scale and exploration of glioma molecular biology which have led to the identification of new therapeutic avenues [2]. Despite these advances, pathophysiologic and translational discoveries have not led to appreciably improved clinical outcomes [3]. Among all glioma subtypes, the glioblastoma multiforme (GBM) is the most aggressive form and commonly results in a poor prognosis [4]. Novel insights into the molecular origins and drivers of GBM progression are needed to enhance our basic understanding and treatment of these tumors.

The ability to look beyond macromolecular changes allowed for the birth of “metabolomics” or an exploration of the oncological roles of small molecule metabolites. In its infancy, metabolomics has shown a promising ability to elucidate metabolic pathway aberrancies involved in various tumor types. One such encouraging discovery was the finding of pathologically related sarcosine accumulation in prostate cancer [5]. Similarly, glycine consumption was linked to tumor proliferation [6]. With respect to glioma, the functional illustration of (R)-enantiomer 2-hydroxygluterate (R-2-HG), produced through gain-of-function isocitrate dehydrogenase-1 (IDH1) mutations has fundamentally changed the way we approach these tumors [7, 8]. R-2-HG has been shown to have multiple oncogenic effects, such as competitive inhibition of the α-ketoglutarate (2KG) binding site of prolyl hydroxylase domain-2 (PHD2) and prevention of hydroxylation and subsequent degradation of hypoxia-inducible factor 1α (HIF-1α) [8]. Related studies have also demonstrated inhibitory effects of R-2-HG on histone demethylation [9]. Clinically, R-2-HG has shown promise as a magnetic resonance spectroscopy-detectable tumor marker [10], offering a paradigm for the translational applications of metabolomics.

Metabolomic analyses of gliomas have been performed by various platforms using a myriad of sample types, with inconsistent and varying results [11–14]. Here, using capillary electrophoresis-mass spectrometry (CE-MS), we identified 16 signature metabolites that are uniquely up or down-regulated in glioma tissues. Hypotaurine was found to have the strongest positive correlation with both GBM occurrence and grade. Taking these findings to an *in vitro* model, we found that hypotaurine enhances glioma cell invasiveness and proliferation. Furthermore, hypotaurine was found to competitively inhibit the 2-KG-binding site on PHD2, resulting in stabilized expression of HIF and its downstream effects. Taurine, the intracellular oxidation product of hypotaurine, repressed intracellular hypotaurine synthesis, resulting in suppression of glioma cell proliferation *in vitro* and arrest of GBM xenograft growth *in vivo*. This data provides new insights into the metabolic underpinnings of GBM proliferation and offers tantalizing new diagnostic and therapeutic targets for this devastating disease.

## RESULTS

### Mass spectrometric profiling of metabolic changes in glioma

**Figure 1:**
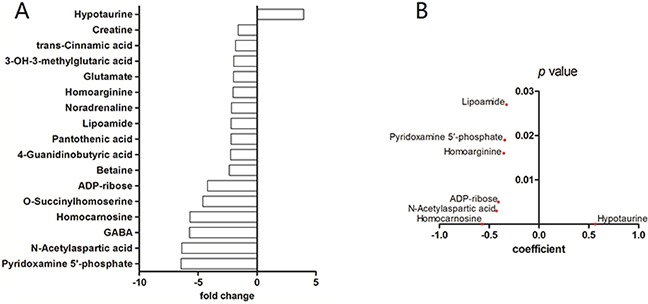
Capillary electrophoresis – mass spectrometry of gliomas **A.** CE-MS of grade-IV gliomas versus control brain tissue. Compounds with significantly different concentrations between the two groups are shown (p<0.05 student's *t*-test). The y-axis displays the varying compounds, and the x-axis represents fold-change in metabolite concentration. **B.** Spearman rank-order correlation for concentration of compounds in (A) across control brains and glioma grades II – IV as determined by CE-MS. The y-axis displays p values and the x-axis shows Spearman coefficient for each compound. Only compounds with coefficient p value less than 0.05 are shown.

CE-MS was performed to investigate the uniquely altered metabolites in GBM. WHO grade-I (n=4), grade-II (n=11), grade-III (n=10), and grade-IV (n=7) glioma specimens were included in the present study. Sixteen areas of grossly normal brain tissue surrounding these tumors and two normal brain sections obtained during surgery for intracranial aneurysms were used as control samples (n=18). Of the 247 metabolites measured, we identified 16 significantly (p<0.05) decreased metabolites, and four increased metabolites in GBM compared to control samples (Supplementary Table S2, Figure 1A). These metabolites were then assessed using a Spearman rank correlation analysis across all tumor grades. Hypotaurine exhibited the strongest positive correlation to tumor grade (p<0.001, Spearman coefficient=0.57, Figure 1B).

### Hypotaurine levels affect the growth characteristics of U251

**Figure 2:**
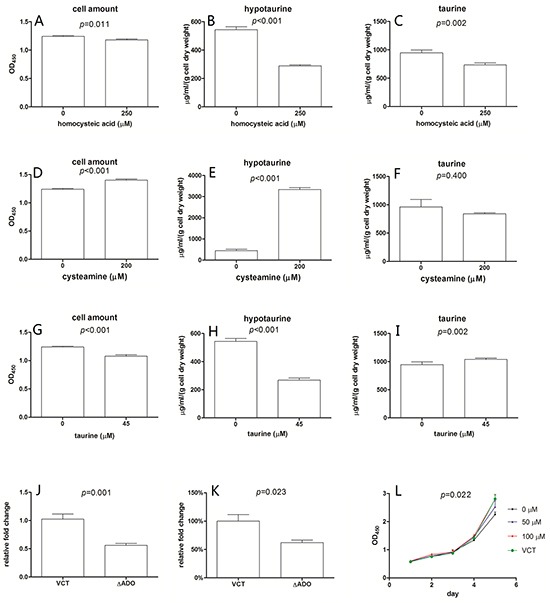
Intracellular content of hypotaurine affects the growth of U251 250μM homocysteic acid resulted in a 5% decrease in cell amount **A.**, a 48% decrease in intracellular hypotaurine **B.** and a 25% decrease in intracellular taurine **C.** Media supplemented with 200μM cysteamine results in a 12% increase in cell amount **D.** a 7-fold intracellular hypotaurine increase **E.** and unappreciable intracellular taurine change **F.** 45μM taurine leads to a 13% decrease in cell amount **G.** a 52% decrease in intracellular hypotaurine **H.** and a 7% increase in intracellular taurine **I.** Cell amount was given as mean±s.d. (n=8) and were determined by OD_450_ after incubated with WST-1. Intracellular hypotaurine and taurine concentrations were given as μg/ml/(g cell dry weight) obtained through at least 3 biological and 3 technical replicates. **J.** Relative ADO gene expression of ADO gene silenced cell line (ΔADO) and the vector control cell line (VCT). A 45% decrease of ADO gene expression in ΔADO cells was observed (n=4). **K.** Compared to VCT cells, intracellular hypotaurine of ΔADO cells decreased by approximately 38% (n=6). **L.** Growth properties of ΔADO cells with different hypotaurine content in the media. VCT cells cultured without additional hypotaurine were used as controls. 100 μM hypotaurine in media led to growth rescue in ΔADO cells (n=8). Data was processed by two-way ANOVA.

Cellular growth properties were investigated in the context of impaired intracellular hypotaurine synthesis (Supplementary Figure S1). Homocysteic acid (HCA) inhibits the activity of cysteinesulfinic acid decarboxylase (EC 4.1.1.29) resulting in suppression of hypotaurine production [15]. We observed that HCA could arrest the growth of U251 cells (Figure 2A), coinciding with the decreased intracellular hypotaurine level (Figure 2B). When U251 cells were cultured in cysteamine, which is converted to hypotaurine by 2-aminoethanethiol dioxygenase (ADO) in astrocytes, cell proliferation was stimulated (Figure 2D) and intracellular hypotaurine levels were increased (Figure 2E) [16]. The conditions mentioned above resulted in decreased intracellular taurine (Figure 2C, 2F). To determine if cell growth was mediated by taurine as well as hypotaurine, we cultured U251 cells in media containing 300% of the normal serum concentration of taurine [16]. In these conditions we observed cell growth arrest (Figure 2G) and decreased intracellular hypotaurine (Figure 2H). Interestingly, there was a corresponding increase in intracellular taurine (Figure 2I). As further confirmation, an ADO gene silenced U251 cell line (ΔADO, Figure 2J) was employed to evaluate the proliferative effects of hypotaurine. The ΔADO cell line had a lower intracellular hypotaurine concentration at baseline (Figure 2K) and a decreased growth rate (Figure 2L). The addition of 100μM hypotaurine rescued the growth characteristics of ΔADO cells. A hypotaurine dose-dependent growth induction was also observed (Figure 2L).

### Molecular analysis of hypotaurine - PHD2 interaction

**Figure 3:**
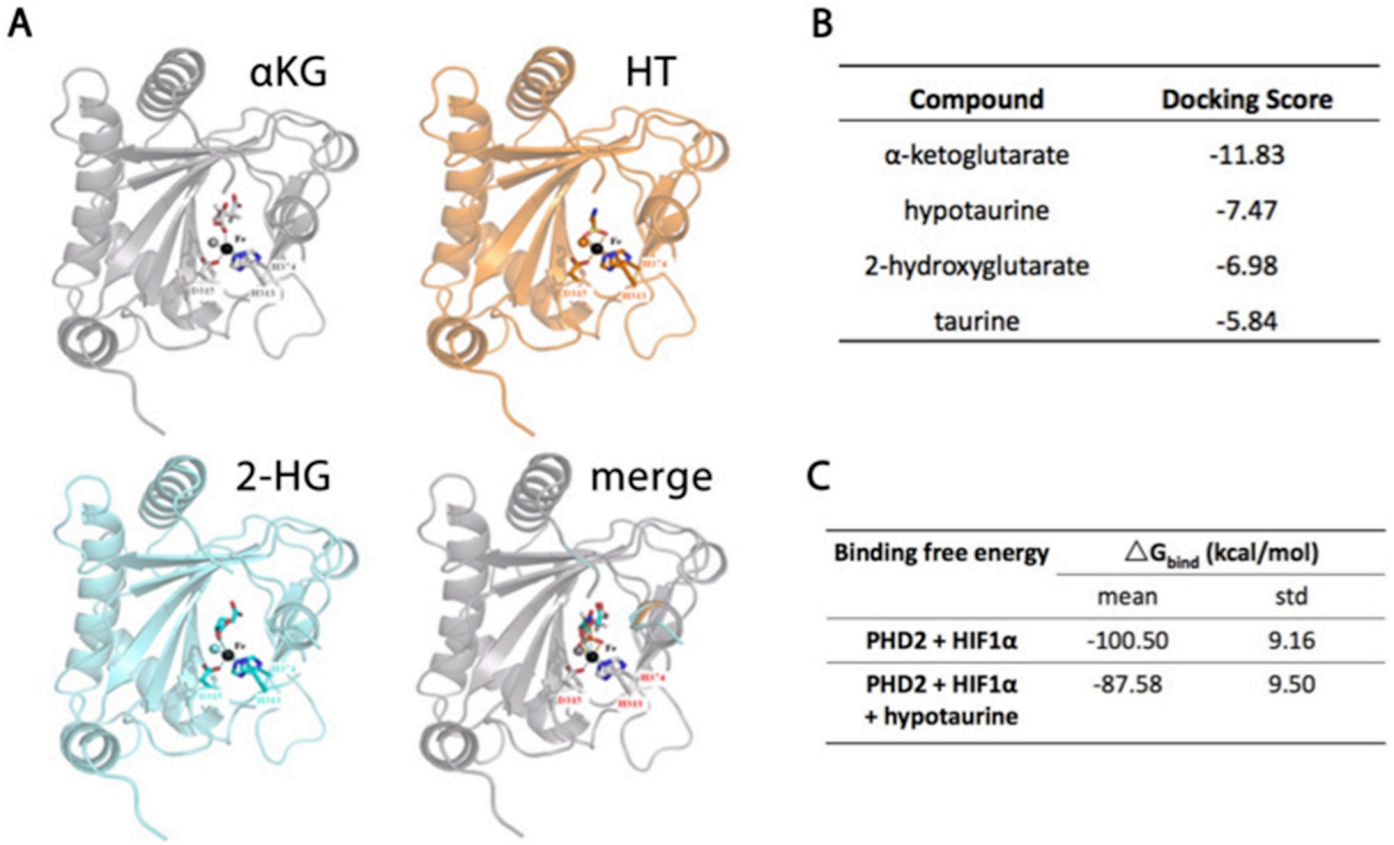
Molecular modeling of hypotaurine, α-KG and 2-HG binding to PHD2 **A.** Ribbon models of PHD2 showing a shared binding site for α-KG, hypotaurine and 2-HG. **B.** Relative docking scores for α-KG, hypotaurine, 2-HG and taurine to the shared binding site on PHD2. A more negative value indicates increased affinity for the binding site. **C.** Binding free energy of PHD2-mediated HIF-1α hydroxylation before and after hypotaurine binding. A more negative value indicates a more spontaneous chemical reaction.

The putative molecular targets of hypotaurine were investigated based on its carbon hydrate structure. *In silico* analysis demonstrated that the hypotaurine binding site on PHD2 with the most favorable energy distribution was the iron-binding interface of H313, D315 and H374 which is shared by 2KG and 2-HG (Figure 3A). Docking scores for 2KG, hypotaurine and 2-HG were calculated and ranked as −11.83, −7.47 and −6.98, respectively (Figure 3B). Calculated binding free energy (ΔG_Bind_) of the HIF-1α-PHD2 interaction was spontaneous at −100.5±9.16 kCal/mol and decreased to −87.58±9.50 kCal/mol in the presence of hypotaurine (Figure 3C).

### HIFα stabilization by hypotaurine through PHD2 inhibition

**Figure 4:**
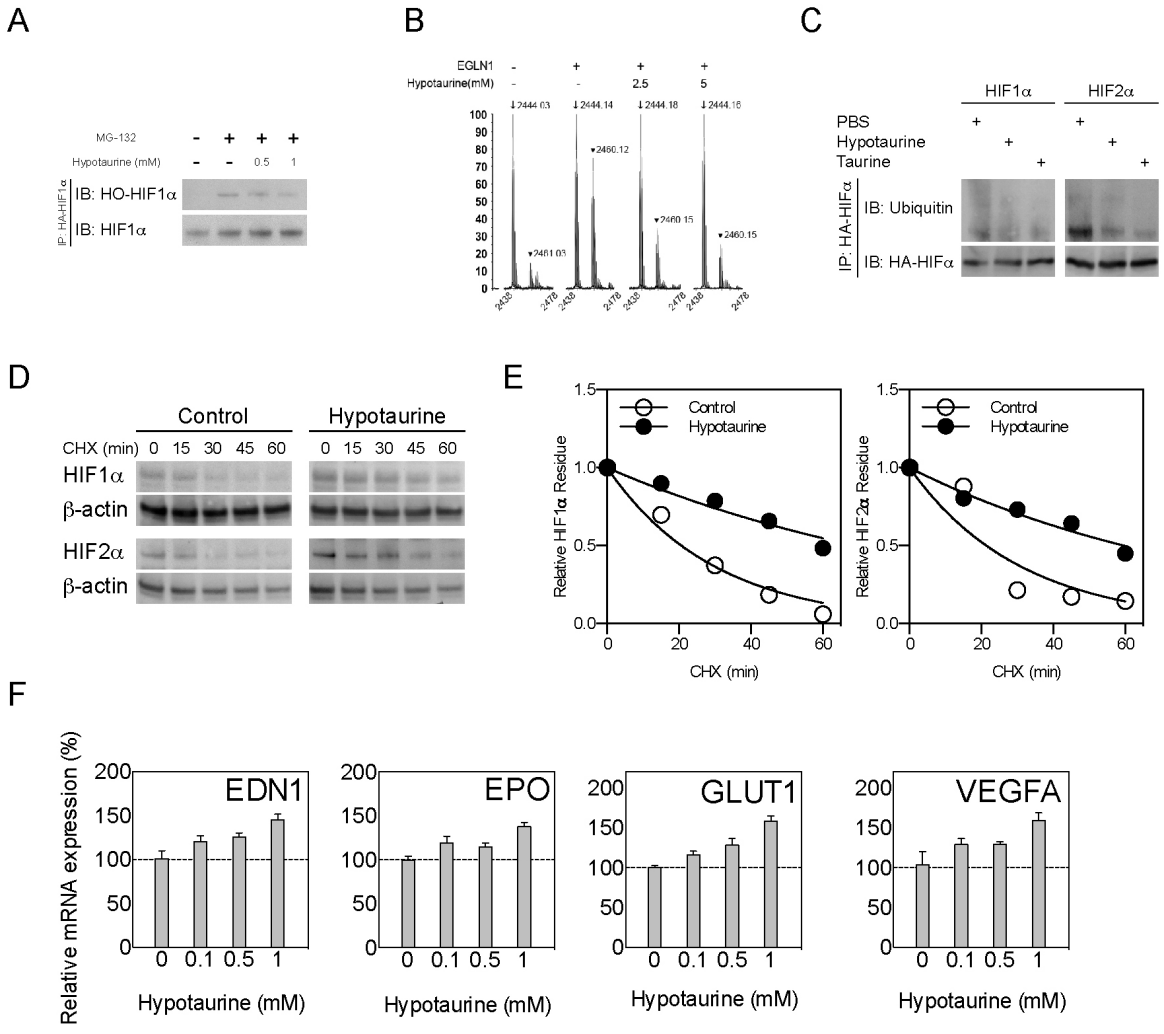
HIF stabilization and HIF pathway activation by hypotaurine **A.** Immunoprecipitation showing hydroxylated (HO-HIF-1α) forms of HIF-1α in glioma cells treated with the proteasome inhibitor MG-132, hypotaurine (0.5 or 1mM), both or neither as indicated. **B.** Hydroxylation assay for HIF-1α after incubation with PHD2 (EGLN1) and 0, 2.5 or 5mM hypotaurine as indicated. The y-axis indicates percentage of maximum signal. The x-axis indicates m/z ratio. Arrows indicate m/z ratio for HIF-1α; arrowheads indicate m/z ratio for OH-HIF-1α. **C.** Immunoprecipitation showing HIF-1/2α ubiquitination in glioma cells treated with PBS or hypotaurine as indicated. **D.** CHX assay of glioma cell cultures treated with PBS or hypotaurine at multiple time-points followed by Western blot for HIF-1α, HIF-2α or β-actin. **E.** Quantification of HIF-1/2α half-lives based on the densiometric analysis of immunoblot in (D). **F.** Real-time quantitative PCR of HIF targets *EDN1*, *EPO*, *GLUT1* and *VEGF* in glioma cells incubated with 0, 0.1, 0.5 or 1mM hypotaurine as indicated.

One of the important molecular functions of PHD2 is to deactivate HIF-α through hydroxylation of the Oxygen Dependent Degradation (ODD) domain [17]. The occupation of hypotaurine in PHD2's catalytic center suggests that it may serve as a competitive inhibitor to PHD2, with consequential abnormal activation of hypoxia signaling. Immunoprecipitation was used to evaluate the hydroxylated form of HIF-1α (HO-HIF1α) in the presence of hypotaurine. Hypotaurine reduced the levels of HO-HIF-1α in U251 cells, with hypotaurine concentrations of 0, 0.5 and 1mM showing qualitative stepwise decreases in HO-HIF-1α (Figure 4A). To confirm the effect of hypotaurine in HIF hydroxylation, we performed an *in vitro* peptide hydroxylation assay as described previously (Figure 4B) [18]. We found that prolyl hydroxylation was attenuated by hypotaurine incubation, with a reduction in hydroxylation of 66.7% at 2.5mM hypotaurine and 82.0% at 5mM hypotaurine, respectively (Figure 4B). Further, we found the protein ubiquitination of HIF-1/2α was reduced in U87 cells treated with 5mM hypotaurine, suggesting a decrease in HIF-1/2α degradation (Figure 4C). Finally, a cyclohexamide (CHX) pulse chase assay demonstrated that hypotaurine extended the half-life of HIF-1α (20.67 min to 69.14 min) and HIF-2α (21.38 min to 59.71 min), indicating hypoxia signaling is activated due to the presence of hypotaurine (Figures. 4D-4E). Moreover, the gene expression of multiple HIF transcriptional targets was increased in a dose-dependent fashion in hypotaurine-treated glioma cells (Figure 4F). Specifically, hypotaurine concentrations of 0.1mM, 0.5mM and 1mM induced an increase in EPO expression to 1.195±0.084 (p>0.05), 1.147±0.05 (p>0.05), and 1.379±0.049, (p<0.05); EDN1 expression to 1.205±0.065 (p>0.05), 1.254±0.05 (p>0.05), and 1.450±0.06 (p<0.01), GLUT1 expression to 1.160±0.043 (p>0.05), 1.281±0.081 (p<0.05), and 1.581±0.067 (p<0.001); and VEGF expression to 1.280±0.075 (p>0.05), 1.292±0.037 (p>0.05) and 1.587±0.098 (p<0.05), respectively.

### Changed cell cycle and invasiveness after hypotaurine treatment

**Figure 5:**
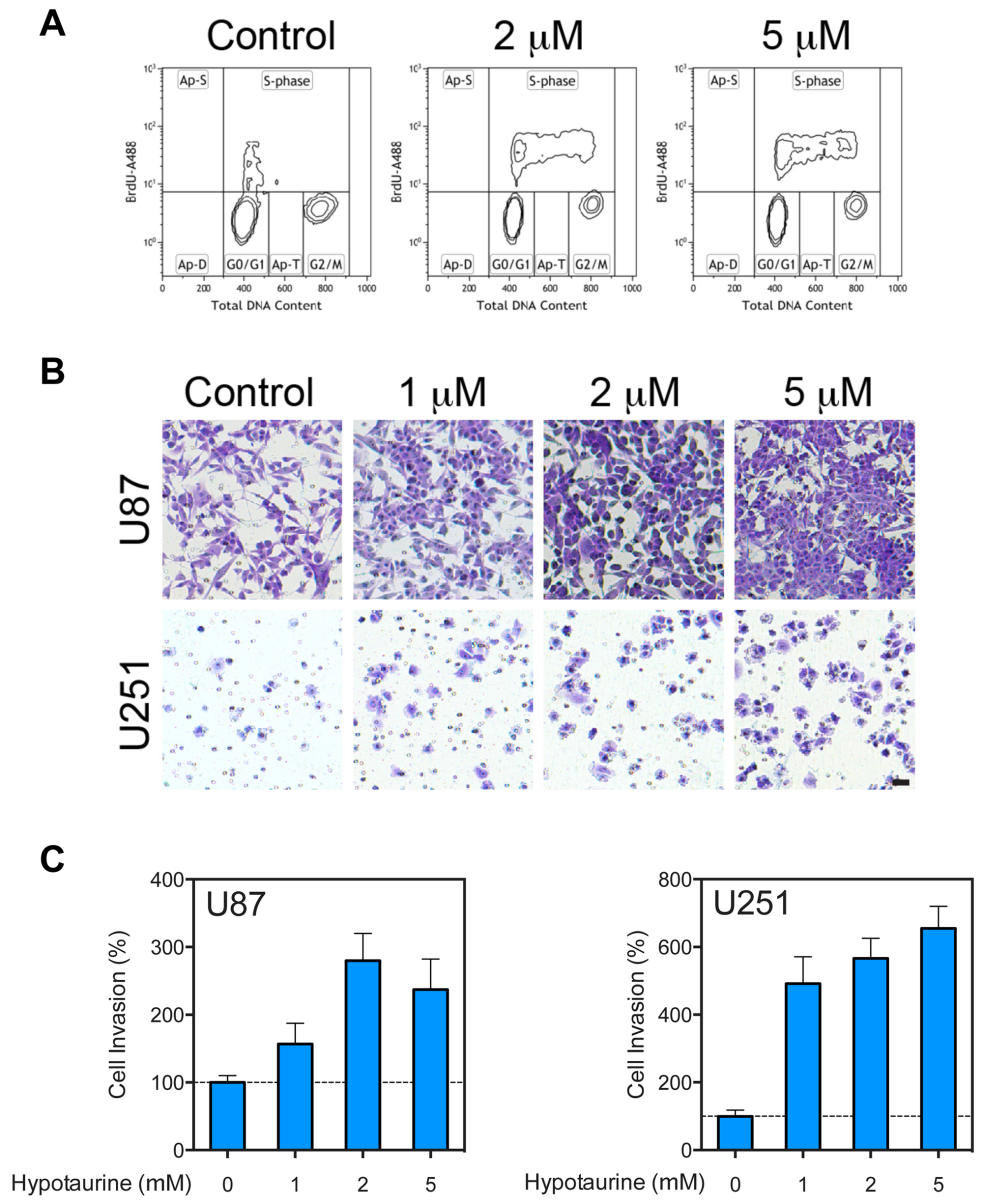
Increased proliferative and migratory capacity of glioma cells treated with hypotaurine **A.** Flow cytometry cell-cycle analysis of glioma cells following treatment with 0, 2 or 5mM hypotaurine. The y*-*axis represents the rate of Brd-U incorporation into DNA on a logarithmic scale. The x*-*axis represents the relative DNA content. **B.** Chemotaxis assay of U87 and U251 glioma cells treated with 0, 1, 2 or 5mM hypotaurine. Cells were plated on the top chamber over a membrane with 8μm pores. Cells that penetrated the membrane were labeled with crystal violet. Scale bar=50μm. **C.** Normalized pixel count for chemotaxis assay of U87 and U251 glioma cells treated with 0, 1, 2 or 5mM hypotaurine. Hypotaurine increased the lower-well cell count in U87 cells by 156.8±30.81% (p>0.05) at 1mM, 279.9±40.26 (p<0.01) at 2mM, and 237.0±45.23% (p<0.05) at 5mM. Similarly chemotaxis was increased in U251 cells relative to control at 1mM by 491.8±79.47% (p<0.001), at 2mM by 566.5±59.28% (p<0.001), and at 5mM by 654.0±65.50% (p<0.001).

To test the hypothesis that hypotaurine induces changes in glioma cell replication and phonotypic mitosis, cell cycle analysis via flow cytometry was utilized to examine changes in mitotic activity. In U87 glioma cells, hypotaurine treatment increased the proportion of cells in S phase from 15.85% to 25.87% at 2mM and 25.30% at 5mM (Figure 5A). In addition, chemotaxis assays revealed that hypotaurine increased the cellular invasion of U87 cells by 156.8±30.81% (p>0.05) at 1mM, 279.9±40.26% (p<0.01) at 2mM, and 237.0±45.23% (p<0.05) at 5mM. Chemotaxis was increased in U251 cells at 1mM by 491.8±79.47% (p<0.001), at 2mM by 566.5±59.28% (p<0.001) and at 5mM by 654±65.5% (p<0.001, Figure 5B and 5C). ADO gene knock down significantly compromised the invasive ability stimulated by cysteamine. On the contrary, cells with intact ADO gene showed an avid cysteamine-induced invasive response (Supplementary Figure S1).

### Increased X_C-_ transporter expression facilitated hypotaurine accumulation

**Figure 6:**
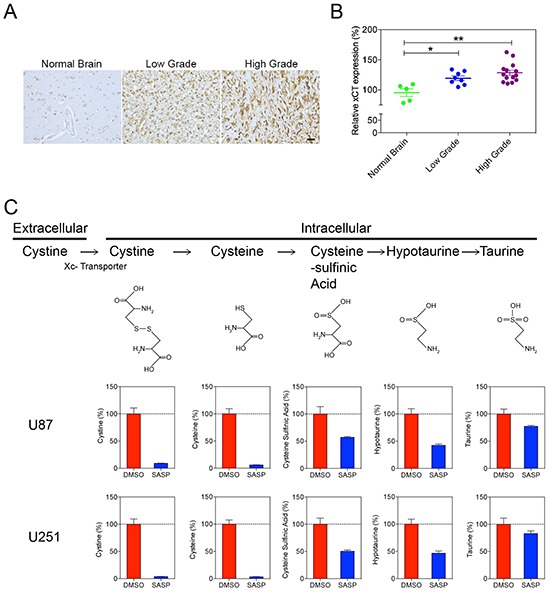
Increased XC- Glutamate-cystine antiporter in high and low grade gliomas **A.** Immunohistochemistry of anti-X_C-_ of normal brain compared to high and low-grade gliomas demonstrates qualitatively increased antiporter staining in glioma tissues. **B.** Image analysis of X_C-_ antiporter staining in low grade glioma (119±10.7, p<0.05) and high grade glioma (129±16, p<0.01). **C.** Isotope labeled metabolite measurement for cystine metabolism. The X_C-_ antiporter inhibitor SASP blocked the cystine metabolism pathway and hypotaurine production in U87 and U251 cells.

For the previously discussed mechanisms of hypotaurine to be functionally relevant, it was necessary to demonstrate mechanisms by which hypotaurine accumulates intracellularly. A likely mechanism for hypotaurine buildup is through the utilization of a cystine/taurine metabolic pathway [16]. Immunohistochemistry studies showed that the X_C_-glutamate-cystine antiporter was highly expressed in human glioma specimens (Figure 6A). Moreover, the differential expression of the X_C_-antiporter positively correlated with the WHO classification of glioma. Quantitative analysis showed that the expression X_C_- antiporter was increased in both low grade (119±10.7%, p<0.05) and high grade (129±16, p<0.01%) gliomas (Figure 6B). Furthermore, the cystine/taurine metabolic pathway was confirmed to be among the major sources of cellular hypotaurine. Isotope labeled metabolite measurement showed that cystine was transported into the cytoplasm (Figure 6C). SASP, an inhibitor of the X_C_^−^antiporter, blocked cystine internalization and consequential hypotaurine production (Figure 6C).

### Taurine ingestion suppressed the growth of U87MG xenografts

**Figure 7:**
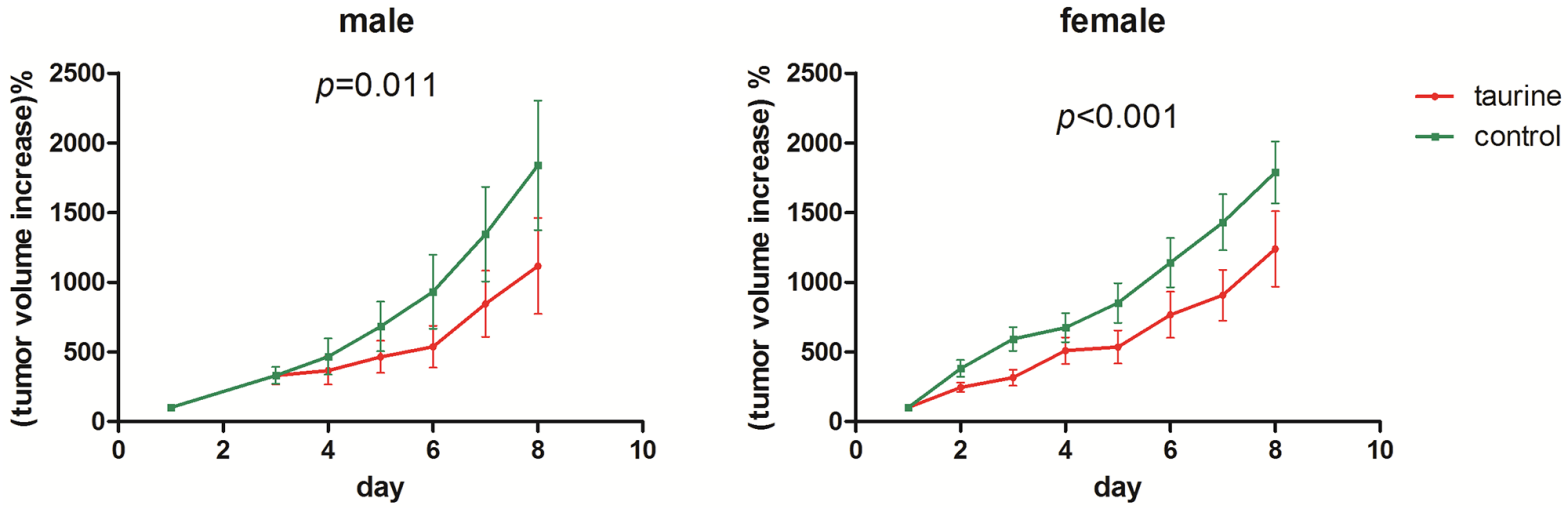
Ingestion of taurine from drinking water could suppress the U87MG xenograft growth in nude mice The y-axis indicates the increased tumor size acquired by volume_day *t*_ - volume_day *0*_. The volume was calculated by (tumor length * tumor width^2^)/2 and volume_day *0*_ was set to 100% for individual tumor. Total 10 pair female and 9 pair male nude mice were enrolled in the experiment.

Our *in vitro* results suggest that taurine can inhibit the synthesis of hypotaurine and arrest the growth of glioma cells (Figure 2G-2I). To verify the importance of this finding in clinically relevant tumors, we examined if taurine might be used to suppress tumor growth *in vivo*. Subcutaneous injections of U87MG were used as an alternative to U251 due to significant technical difficulty in the formation of solid U251 implantation tumors in the selected nude mice (data not shown). During the experimental period, matched eligible mice were surveyed from each group. All tumors grew significantly during the observation period (p<0.001) for both groups (Figure 7). Treatment with 1% taurine water resulted in pronounced growth delay of the implanted tumors (Figure 7). This result demonstrates that ingestion of taurine correlated with a reduced rate of tumor growth.

## DISCUSSION

In this paper, we aimed to identify aberrant metabolic pathways in gliomas and their effects on tumor cellular behavior. Using capillary electrophoresis – mass spectrometry, we identified hypotaurine as a biomarker of glioma presence, with a strong positive correlation between hypotaurine level and glioma grade. Little is currently known about the biological role of hypotaurine. Multiple groups have identified hypotaurine in murine glioma cell lines and in the tumor bed of intracranial glioblastoma xenografts [19–21]. Our study is the first to demonstrate the presence of hypotaurine in human brain tumors and to correlate hypotaurine levels with glioma grade. Although grade I glioma is considered to be biologically distinct from higher grade gliomas, we found its inclusion in this study necessary to elucidate fundamental metabolic changes occurring in glioma that may affect general tumor growth characteristics. These findings suggest that hypotaurine is present in multiple types of glioma and is largely confined to the tumor core.

Taurine content in normal astrocytes is relatively constant and, as confirmed in this study, is not altered by primary substrate supplementation [16]. Taurine is elevated in various neoplastic tissues and may be associated with susceptibility to chemotherapy [19, 22–25]. Increased taurine synthesis has been postulated as a cellular response to ischemia and reperfusion injury. Ischemia with subsequent reperfusion leads to accumulation of coenzyme A, a key substrate for taurine synthesis [26]. Hypotaurine levels are also elevated in hypoxia and provide antioxidant protection upon reperfusion [27]. Taurine supplementation decreased intracellular hypotaurine concentrations, implying that extracellular taurine availability might not initiate de novo hypotaurine synthesis. Studies have found low taurine levels in glioma tissue and here we show that changes in taurine concentration can drastically alter intracellular hypotaurine levels [28]. Also, while high hypotaurine did not contribute to intracellular taurine, low hypotaurine content did compromise taurine synthesis. A possible mechanism for increased hypotaurine levels in glioma may be due to the increased presence of the X_C-_ antiporter compared to normal brain. We speculate that variations in intracellular hypotaurine levels imply a functional role to the metabolite beyond that of antioxidation to one of oncometabolite. This is supported by our finding that hypotaurine participates in cell cycle regulation, promoting cellular proliferation and invasion. The observed invasiveness is likely mediated by the stabilizing effects of hypotaurine on the HIF-mediated signaling cascade via hypotaurine's competitive inhibition of PHD2.

Hypoxia and alterations of hypoxic signaling, such as that demonstrated by hypotaurine on HIF-1α, have been well described as mechanisms of cancer development and growth. Intratumoral hypoxia has, in fact, been studied comprehensively in glioma. Intra- and peri-tumoral oxygenation has been recorded in GBM patients to show that intratumoral tissue is significantly less oxygenated than peri-tumoral regions [29]. Hypoxia has also been shown to induce dedifferentiation and preservation of stem-like cells in glioma [30, 31]. Moreover, the HIF signaling pathway is critical to the tumorigenic and invasive phenotype of glioma, contributing to the chemo and radioresistance of tumor cells [32, 33]. Although HIF-1α has been targeted by various therapies, the results in patients with GBM are limited [34, 35].

Under normoxic conditions, proline residues on HIF-1α are hydroxylated by PHD2 in an oxygen-dependent fashion, thereby targeting HIF-1α for ubiquitination and proteosomal degradation [26, 27]. Under hypoxic conditions, the HIF α subunit forms a heterodimer with a constitutively expressed HIF β subunit in the nucleus, forming a transcriptionally active complex, and leading to up-regulation of a myriad of angiogenic and proliferative factors such as VEGF, EPO, GLUT1, and EDN1 [28, 36]. Many tumor types, such as RCC, and those tumors associated with von-Hippel Lindau (VHL) syndrome create “pseudohypoxia” via inactivating mutations of the VHL protein that would normally ubiquitinate HIF-1α. This pseudohypoxia allows for stable transcription of the HIF targets and confers a hyperproliferative and invasive phenotype associated with an increase in glycolytic metabolism, angiogenesis and markers of epithelial-to-mesenchymal transition [37–42]. Notably, pseudohypoxia through HIFα stabilization in malignant glioma is a known consequence of IDH1 R132H mutation status [43, 44]. Our data supports this finding, demonstrating that hypotaurine acts to both potentiate HIF-1α stability and signaling as well as confer an invasive phenotype consistent with pseudohypoxia in tumor models. Thus, our data coincides with the current understanding of metabolic alterations in pseudohypoxic signaling in gliomas and provides an oncometabolite-mediated mechanism that parallels the HIFα stabilization known to be present in IDH-mutant gliomas.

We were also able to target hypotaurine *in vivo* by demonstrating that taurine consumption decreased tumor growth. Although taurine's physical properties prevent it from freely crossing the blood-brain barrier (BBB), it has been extensively studied in the CNS for its antioxidant, anti-inflammatory, neuroinhibitory and neuroprotective effects in various disorders, such as Alzheimer's and Parkinson's disease [45]. As the enzyme responsible for taurine synthesis is low and rate-limiting in cats, primates, and humans, dietary taurine is responsible for maintaining taurine levels within the body [45, 46]. Dietary taurine enters the brain through transport across the TauT specific taurine transporter at the BBB [47]. Our *in vivo* data showed that taurine supplementation led to increased intracellular taurine, decreased intracellular hypotaurine and concomitant inhibition of cell growth was also demonstrated *in vivo*.

No genetic analysis was performed in this study, but the possible link between oncogenetic changes and hypotaurine elevation is an intriguing one that requires further investigation. The growing understanding of metabolomics has begun to shed light on the interaction between well-delineated oncogenic pathways and newly described metabolic alterations. This data offers an example of our increased understanding of the interplay between biochemical metabolism, signal transduction, and tumor behavior. We show that hypotaurine enhances glioma cell proliferation and invasion and offers a potential target for biomarker-enhanced diagnosis, staging and therapy. Further investigation into the oncogenic effects of hypotaurine and the cause of its elevation in human cancer is necessary in order to provide a meaningful translational tool in glioma treatment.

## MATERIALS AND METHODS

### Human tissue analysis

This study was approved by the Medical Committee of Ethical Experiments at The First Affiliated Hospital of Harbin Medical University and informed consent was obtained from all participants. Human tissue samples were collected from The First Affiliated Hospital of Harbin Medical University (Supplementary Table S1). Tumor specimens included in the analysis were 11 WHO grade-II gliomas (two astrocytic, seven oligodendroglial, seven astrocytic mixed with oligodendrocytic components), 10 grade-III gliomas (three oligodendroglial, seven astrocytic mixed with oligodendrocytic components) and seven grade-IV GBM. The 18 control samples were collected from grossly normal brain adjacent to resected tumor tissue (two WHO grade-I glioma, six grade-II glioma, three grade-III glioma, four grade-IV glioma and one central neurocytoma) or two operable cerebral aneurysms. All surgical specimens were immediately stored at−80°C. The CE-MS protocol is fully described in the Supplementary Information. Briefly, sample preparation was based on protocols provided by Human Metabolome Technologies, Inc. (HMT; Tokyo, Japan). CE-MS was performed on an Agilent 7100 capillary electrophoresis system coupled to a 6224 time-of-flight liquid chromatography mass spectrometry (TOF-LC/MS) instrument (Agilent Technologies, Santa Clara, CA). CE-MS data were collected using Agilent Mass Hunter Ver. B.04.00software (Santa Clara, CA). Peak picking and metabolite identification were performed in accordance with the standard protocol provided by HMT. Zero values in the data were removed according to the 80%-rule as proposed [37]. A student's t-test and Spearmann rank-order correlation for human sample data were performed using MINTAB V.16 (State College, PA) and results were considered significant if *P*<0.05.

### Quantitation of intracellular taurine and hypotaurine

Cells were cultured in 10cm plate with total medium volume of 10 ml. Nearly 1–5×10^6^ cells were seeded for each plate. Different concentrations of reagents were added to disturb the intracellular concentrations of hypotaurine and taurine as necessary (Supplementary Figure S1). The cells were washed with cold phosphate buffered saline (PBS) in triplicate and 1ml cold water and methanol (v/v 79:21) were evenly spread onto each plate. The adherent cells were collected using a cell scraper and each sample was transferred into a 15ml centrifugation tube containing 2ml ice-cold chloroform. The mixture was sonicated briefly and then centrifuged in 4°C for 15 min at 12000g. Every 700μl supernatant was pipetted out and lyophilyzed at −50°C in a Labconco freeze dry system (Kansas City, MO). The protein layer for each sample was collected to dry until constant weight for intracellular taurine and hypotaurine was achieved. The concentrations were then normalized. For hypotaurine and taurine quantitation, an AccQTag Ultra Derivatization Kit from Waters (Milford, MA) was used. Every lyophilized sample was dissolved in 20μl methanol and water (21:79). 10μl of the mixture was used for derivatization according to the instructions provided by the manufacturer. Liquid chromatography separation was performed on a Waters ACQUITY Ultra performance LC system using a BEH C18 column (2.1mm×100mm, 1.7μm). Mobile phase A was 20mM ammonium formate dissolved in 0.5% formic acid and 1% acetonitrile water solution. Phase B was 1.6% formic acid dissolved in pure acetonitrile. The elution gradient of phase B was: 0-1.08min, 0.1% B; 1.08-11.48 min,0.1% −9.1% B; 11.48-16.3, 9.1%-21.2%B; 16.9-18.1 min, 59.6% B; 18.28-20min, 0.1%B. the flow rate was 0.35mL/min. The injection volume was 1μL. The column temperature was 55°C with sampler temperature of 15°C. Mass spectrometry analysis was accomplished on a Waters ACQUITY SQ Detector system scanned in positive SIR mode. The capillary voltage was 3 kV. Cone voltage was 30eV. The desolvation and cone temperature was 300°C and 120°C separately. The desolvation gas flow was 650L/h and cone gas flow was 50L/h.

### Isotope labeled metabolite detection

Glioma cells of U251 and U87 were cultured in 10cm dishes using Dulbecco's modified eagle medium (DMEM), high glucose, supplemented with 10% fetal bovine serum. The cells were first cultured at 37°C in a full humidity incubator for 24h with 5% CO_2_. After that, DMSO dissolved sulfasalazine (SASP) was added to some dishes at the final concentration of 0.5mM. As control, the other dishes were added with the same volume of DMSO. After another 24h culture, isotope labeled L-cystine (13C6,99%; 15N2, 99%, Cambridge Isotope Laboratories, Inc., Andover, MA) were added to each dish with the final concentration of 100μM. The cells were cultured for another 6h. For each cell type of each treatment, total 6 replicates were prepared. The cells were collected by sequential washing with large volume of PBS and 1ml of 21:79 methanol/water. The cells suspension was mixed with 2ml of chloroform and sonicated briefly for metabolite extraction. Every 700μl of supernatant was precisely allocated to a 1.5ml tube after centrifugation at 12000×g. All the extracts were lyophilyzed at −50°C in a Labconco freeze dry system (Kansas City, MO). AccQTag Ultra Derivatization Kit from Waters (Milford, MA) was used for sample derivatization. Every lyophilized sample was dissolved in 80μl methanol and water (21:79). 20μl derivatization reagent was added and incubated at 55°C for 10min. Liquid chromatography separation was performed on an Angilent 1290 Infinity ultra performance LC system using an Agilent C18 column (2.1mm×100mm, 1.8μm). Mobile phase A was 5mM ammonium formate dissolved in 0.5% formic acid water solution. Phase B was pure acetonitrile. The elution gradient of phase B was: 0-1.0min, 5% B; 1.0-20 min,5% −25% B; 20.0 −25.0, 25%-57%B; 25.1-27.0 min, 99% B; 27.1-30min, 5%B. The flow rate was 0.25mL/min. The injection volume was 1μL. The column temperature was 50°C. Mass spectrometry analysis was accomplished on an Agilent 6460 Triple Quad LC/MS system equipped with an ESI source and scanned in positive multiple reaction mode (MRM). The collision energy was 20 kV. The fragmentor voltage was 120V (for cysteine, hypotaurine and taurine detection) or 135V (for cystine and cysteinesulfinic acid detection)

### In silico modeling of hypotaurine binding to PHD2

For molecular simulation studies, the binding pocket was defined by the position of the small molecule in the crystal structure of PHD2 (PDB code 3HQR) [43]. Q-Site Finder (University of Leeds, Leeds, UK) was used to identify all possible binding pockets for α-KG, hypotaurine, and R-2-HG in PHD2 [44]. Glide V5.5 (Schrödinger, Inc., New York, NY) was employed to dock substrates to PHD2 [48]. Electrostatic potentials were derived using the RESP charge fitting by antechamber module in the Amber 12 package [49]. Each PHD2 complex bound to α-KG, hypotaurine, 2-HG and HIF1α was solvated in a periodic box of TIP3P water model [50]. Energy minimization and MD simulations were performed by PMEMD module of Amber 12 [51]. The SHAKE algorithm was adopted to constrain all bonds involving hydrogen [51]. The particle mesh Ewald method was applied to treat the long range electrostatic interactions [52, 53]. Binding free energies between HIF1α and PHD2 were calculated using the molecular mechanics Poisson-Boltzmann surface area method [54].

### Cell culture

U251 and U87 cells (American Type Culture Collection) were cultured in DMEM with 10% FBS at 37°C with 5% CO2 in a humidified incubator with or without MG-132 or hypotaurine (Millipore, Billerica, Massachusetts) as indicated. For growth analysis, cells were seeded in 96-well plates with each well containing 10^3^∼10^4^ cells/ml. After 24hr, 20μl of WST-1 (Roche Diagnostics, Indianapolis, IN) was added to each well and the plates were incubated for an additional 3hr. Plates were read by a Bio-Rad 550 ELISA reader (Hercules, CA) with a working wavelength of 450nm.

### Immunoprecipitation and western blot

Cell pellets were lysed using a RIPA lysis buffer with Halt proteasome inhibitor (Thermo Scientific), sonicated briefly and centrifuged at 15000 × g at 4°C for 15 minutes. Immunoprecipitation was performed as described previously [55]. Antibodies against HA-HIF1α were obtained from Covance (Princeton, New Jersey). An indirect immunoprecipitation kit (Millipore) and manufacturer-suggested protocols were used. Immunoblot was performed as described previously [56]. PVDF Membranes were probed using antibodies to HA-HIF1α (Covance), HO-HIF1α (Cell Signalling Technology, Danvers, Massachusetts), HIF1α (Novus Biologicals, Littleton, Colorado), HIF2α (Novus) or ubiquitin (Abcam, Cambridge, Massachusetts).

### HIF hydroxylation assay

Peptide hydroxylation assay was performed as previously described [18, 57].

### Cycloheximide chase assay

Cycloheximide assay was performed as described previously [58]. Protein extract was subjected to immune blot for HIF1α (Novus) or HIF2α (Novus) and α-actin (Santa Cruz Biotechnology, Dallas, Texas) as described above.

### Quantitative real-time PCR

Total RNA was isolated from tumor samples using an RNeasy kit (Qiagen) or RNAiso plus (TaKaRa, Dalian, China). Messenger RNA expression was quantified using an Illumina Eco Real-Time PCR System (Illumina, Inc., San Diego, CA) using primers for Endothelin-1 (*EDN1)*, Erythropoietin (*EPO)*, Glucose Transporter 1 (*GLUT1)* and Vascular Endothelial Growth Factor A (*VEGFA)* (Qiagen, Venlo, Limburg, Netherlands) or Mx3000P Real Time PCR System (Agilent, Santa Clara, CA) using primers (5′-GGAGCACTGTTTCTCCCTTTT-3′ and 5′-CAATCAAGAGGGCTTAGACGA-3′) for 2-aminoethanethiol (cystamine) dioxygenase (ADO) gene.

### Flow cytometry cell cycle assay

Cell cycle analysis was performed as previously described [59].

### Transwell invasion assay

Fifty thousand U87 or U251 cells were suspended in serum-free medium and plated to the upper chamber of Transwell inserts (BD Biosciences, Bedford, Massachusetts). Medium with 10% FBS was added to the lower chamber. Cells were incubated for 12 hours and non-migrated cells were removed. Cells on the lower surface were stained with crystal violet. Cell morphology was recorded by light microscope with magnification of 20x. Each condition was analyzed in triplicate. For quantitative analysis invasion ability of ΔU251 and its vector cells, QCM Cell Invasion Assays kits from Millipore (Shanghai, China) were selected. All the performance was conducted according to the instructions provided by the manufacturer. Fluorescent intensity was detected by Cary Eclipse from Agilent (Santa Clara, CA). The excitation and emission wavelengths were 480 and 520nm respectively. Each condition was analyzed in triplicate.

### Animal experiment

All the experiments were performed with the approval and survey of The Animal Ethics Committee of Dalian Medical University. BALB/c nu-nu SPF nude mice of age 4 to 6 weeks were provided by Experimental Animal Center of Dalian Medical University. Tumor burden models were constructed by subcutaneous injection of U87MG cells in the right shoulder of each mouse. Briefly, 1 ml cell pellet (∼10^8^ cells) was mixed with equal volume of Matrigel basement membrane matrix (BD Biosciences, San Jose, CA) in an ice bath. 50ul of the mixture was injected in each mouse. After 1 week, 40 tumor burden mice with comparable tumor sizes were randomly divided into test and control groups, and matched for gender across groups. All the mice were housed in an aseptic environment with free access to aseptic water and diet. When the mice were divided into different groups (day 0), aseptic water with final concentration of 1% taurine was given only for the test groups. The room temperature was kept at 25°C with a day-night cycle of 12 hours. Water and diet were replenished every other day. Each tumor diameter (length and width) was measured on day 0. In the following experiment days, tumor size (length × width^2^/2) for each mouse was recorded against their corresponding day 0 volume. The increased volumes were compared by ANOVA using Minitab V.16 (Minitab Inc., State College, PA) between the two groups.

## SUPPLEMENTARY MATERIALS FIGURE AND TABLES




