# Supplementary material for: Hypotaurine evokes a malignant phenotype in glioma through aberrant hypoxic signaling
Source: Oncotarget. 2016 Feb 25;7(12):15200–14. doi: 10.18632/oncotarget.7710 (PMC4924780; doi:10.18632/oncotarget.7710)
Supplement: Supplementary file 1 [file oncotarget-07-15200-s001.pdf]

SUPPLEMENTARY MATERIALS AND METHODS

Reagents

Homocysteic acid (HCA), cysteamine, hypotaurine, taurine were all purchased from Sigma-Aldrich (Shanhai, China).

Cell culture

U251 cells were cultured in DMEM high glucose media with 10% fetal bovine serum at 36.5°Cwith 5% CO_2 in a fully humidified incubator. For growth property analysis, cells were seeded in 96-well plates with each well containing 200µl of ~10^4/ml cells. After 24h, every 20µl of WST-1(Roche Diagnostics, Indianapolis, IN) was added to each well and the mixture was incubated for another 3h in the above mentioned environment. The plates were read by a BIO-RAD 550 ELISA reader (Hercules, CA) with the working wavelength of 450nm.

The cell line of 2-aminoethanethiol dioxygenase (ADO) gene stable knockout U251 (ΔADO) and its vector counterpart were purchased from 3DBiopharm (Shanghai, China). These cells were cultured at the presence of 0.5 µg/ml puromycin in DMEM high glucose media containing 10% fetal bovine serum.

Culture condition of U87MG cells was identical to that of the U251 cells.

REFERENCE

1. Vitvitsky,V., Garg,S.K., & Banerjee,R. Taurine biosynthesis by neurons and astrocytes. J. Biol. Chem. 286, 32002-32010 (2011).

SUPPLEMENTARY FIGURE AND TABLES

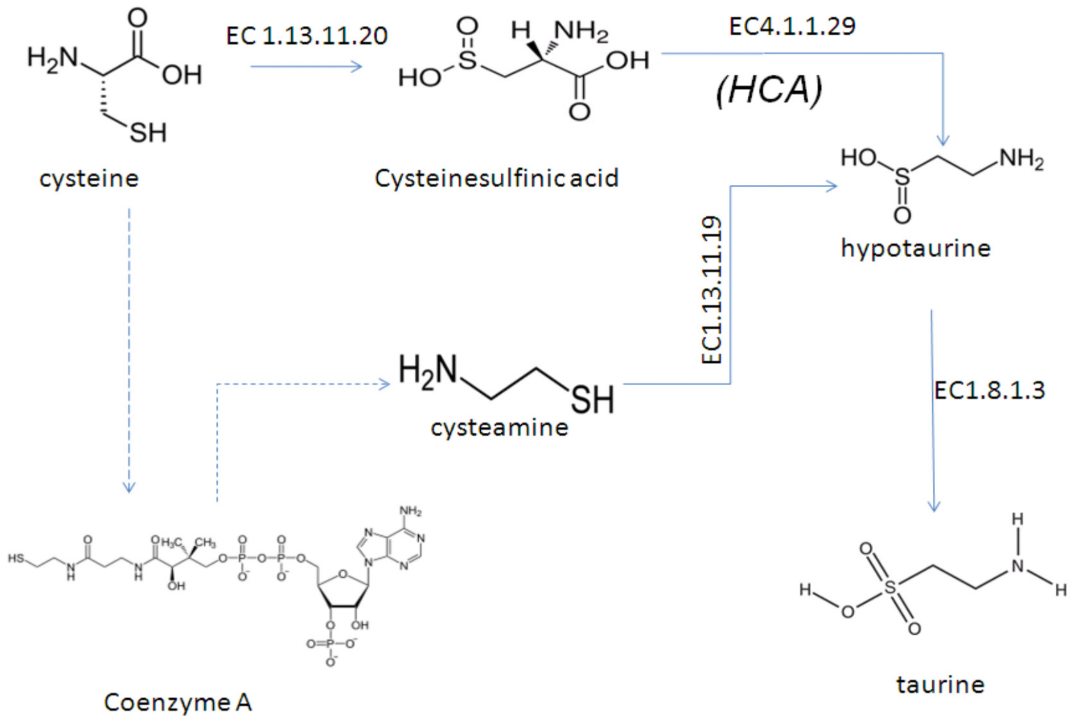

Supplementary Figure S1: Schematic representation of hypotaurine biosynthesis pathways in the cell[1].

Supplementary Table S1: Sample Information

Tumor Type	WHO grade	total
Tumor adjacent tissue from Grade II astrocytoma	control	
Tumor adjacent tissue from Grade I glioma	control	
Tumor adjacent tissue from Grade II astrocytoma	control	
Tissue from aneurysm decompression operation	control	
Tissue from aneurysm decompression operation	control	
Tumor adjacent tissue from Grade IV glioblastoma	control	
Tumor adjacent tissue from Grade II astrocytoma	control	
Tumor adjacent tissue from Grade II astrocytoma	control	
Tumor adjacent tissue from glioblastoma multiforme	control	
Tumor adjacent tissue from Grade II astro-mesoglioma	control	
Tumor adjacent tissue from Grade III astrocytoma	control	
Tumor adjacent tissue from glioblastoma multiforme	control	
Tumor adjacent tissue from Grade I - II astrocytoma	control	
Tumor adjacent tissue from Grade III astrocytoma	control	
Tumor adjacent tissue from anaplastic oligodendroglioma	control	
Tumor adjacent tissue from central neurocytoma	control	
Tumor adjacent tissue from glioblastoma multiforme	control	
Tumor adjacent tissue from Grade II - III glioma	control	18
Oligodendroglioma	II	
Astro-oligodendroglioma	II	
Oligodendroglioma	II	
Astro-oligodendroglioma	II	
Astro-oligodendroglioma	II	
Astrocytoma	II	
Astro-oligodendroglioma	II	
Astro-oligodendroglioma	II	
Astro-oligodendroglioma	II	
Astro-oligodendroglioma	II	
Glioma	II	11
Anaplastic oligodendroglioma	III	
Anaplastic oligodendroglioma	III	
Glioma	III	
Glioma	III	
Glioma	III	
Glioma	III	

(*Continued*)

Tumor Type	WHO grade	total
Glioma	III	
Glioma	III	
Glioma	III	
Anaplastic oligodendroglioma	III	10
Glioblastoma multiforme	IV	
Glioblastoma multiforme	IV	
Glioblastoma multiforme	IV	
Glioblastoma multiforme	IV	
Glioblastoma multiforme	IV	
Glioblastoma multiforme	IV	
Glioblastoma multiforme	IV	7

Supplementary Table S2: Identified metabolites by CE-MS analysis

(See Supplementary File 1)
