# Supplementary material for: Hypotaurine evokes a malignant phenotype in glioma through aberrant hypoxic signaling
Source: Oncotarget. 2016 Feb 25;7(12):15200–14. doi: 10.18632/oncotarget.7710 (PMC4924780; doi:10.18632/oncotarget.7710)
Supplement: Supplementary file 2 [file oncotarget-07-15200-s002.docx]

Table S2 Identified metabolites by CE-MS analysis

| m/z | Corrected Migration Time | Candidate metabolite detected in positive mode | m/z | Corrected Migration Time | Candidate metabolite detected in negative mode |
| --- | --- | --- | --- | --- | --- |
| 61.0396 | 20.644 | Urea | *73.0295* | *11.033* | *Propionic acid* |
| 74.0964 | 7.038 | Isobutylamine | *85.0295* | *10.361* | *Crotonic acid* |
| 75.0917 | 4.381 | 1,3-Diaminopropane | *87.0452* | *9.866* | *Butyric acid or Isobutyric acid* |
| 76.0757 | 6.642 | Trimethylamine N-oxide | *88.004* | *13.263* | *Oxamic acid* |
| 76.0757 | 6.845 | Isopropanolamine | *89.0244* | *10.8* | *Lactic acid* |
| 80.0495 | 5.873 | Pyridine | *101.0244* | *9.978* | *Succinic semialdehyde* |
| 81.0447 | 13.55 | Pyrimidine | *101.0244* | *11.382* | *2-Oxobutyric acid* |
| 86.0964 | 7.177 | Piperidine | *101.0608* | *9.263* | *Valeric acid* |
| 100.1121 | 7.694 | Cyclohexylamine | *103.0037* | *30.858* | *Malonic acid* |
| 102.055 | 7.122 | 1-Aminocyclopropane-1-carboxylic acid | *105.0193* | *10.433* | *Glyceric acid* |
| 102.0913 | 7.388 | Betaine aldehyde | *110.9758* | *16.05* | *Methyl sulfate or Methyl sulfate* |
| 103.123 | 4.933 | Cadaverine | *111.0088* | *10.695* | *2-Furoic acid* |
| 104.0706 | 7.547 | GABA | *115.0037* | *25.156* | *Fumaric acid* |
| 104.0706 | 9.639 | 2-Aminoisobutyric acid | *115.0765* | *8.952* | *Hexanoic acid* |
| 104.0706 | 10.242 | N-Methylalanine | *117.0193* | *20.648* | *Methylmalonic acid* |
| 104.0706 | 10.947 | N,N-Dimethylglycine | *117.0193* | *20.944* | *Succinic acid or Methylmalonic acid* |
| 110.027 | 17.927 | Hypotaurine | *121.0295* | *9.961* | *Benzoic acid* |
| 110.06 | 7.615 | o-Aminophenol | *128.0353* | *9.573* | *5-Oxoproline* |
| 112.0869 | 4.766 | Histamine | *129.0557* | *9.14* | *5-Oxohexanoic acid* |
| 115.0502 | 21.298 | 1-Methylhydantoin | *129.0557* | *9.646* | *4-Methyl-2-oxovaleric acid or 3-Methyl-2-oxovaleric acid* |
| 118.0611 | 8.231 | Guanidoacetic acid | *130.051* | *8.924* | *N-Acetyl-beta-alanine* |
| 118.0863 | 8.003 | 5-Aminovaleric acid | *131.035* | *16.701* | *Glutaric acid or Glutaric acid* |
| 118.0863 | 10.023 | Val | *131.0462* | *9.327* | *3-Ureidopropionic acid* |
| 118.0863 | 11.254 | Betaine | *131.0714* | *8.682* | *2-Hydroxy-4-methylvaleric acid* |
| 120.0655 | 7.938 | 4-Amino-3-hydroxybutyric acid | *135.0299* | *9.313* | *Threonic acid* |
| 120.0655 | 10.108 | Homoserine | *135.0452* | *9.231* | *p-Toluic acid* |
| 120.0655 | 10.384 | Thr or 2-Methylserine | *137.0244* | *9.162* | *m-Hydroxybenzoic acid* |
| 121.0509 | 8.081 | Purine | *137.0244* | *9.375* | *p-Hydroxybenzoic acid or m-Hydroxybenzoic acid* |
| 123.0553 | 7.394 | Nicotinamide or Isonicotinamide | *137.0244* | *10.483* | *o-Hydroxybenzoic acid* |
| 126.0219 | 21.318 | Taurine | *140.0118* | *8.003* | *Ethanolamine phosphate* |
| 126.0662 | 7.622 | 5-Methylcytosine | *141.0193* | *18.266* | *cis£¬cis-Muconic acid* |
| 130.0863 | 10.263 | Pipecolic acid | *147.0299* | *17.295* | *Citramalic acid* |
| 130.159 | 8.414 | Octylamine | *147.0452* | *8.786* | *trans-Cinnamic acid* |
| 131.1179 | 8.48 | N-Acetylputrescine | *151.0262* | *8.015* | *Xanthine* |
| 131.1291 | 5.082 | Agmatine | *151.0401* | *8.911* | *Mandelic acid or p-Anisic acid or p-Hydroxyphenylacetic acid* |
| 132.0655 | 12.007 | trans-4-Hydroxyproline or Hydroxyproline | *151.0401* | *9.162* | *Phenoxyacetic acid or Mandelic acid or p-Anisic acid* |
| 132.0768 | 8.808 | Creatine | *152.0023* | *9.546* | *Cysteinesulfinic acid* |
| 132.1019 | 8.288 | 6-Aminohexanoic acid | *153.0193* | *9.575* | *2£¬5-Dihydroxybenzoic acid* |
| 132.1019 | 8.667 | beta-Leucine | *155.0098* | *9.697* | *Orotic acid or 2£¬4-Dihydroxypyrimidine-5-carboxylic acid* |
| 132.1019 | 10.213 | Leu or Ile | *157.1234* | *7.979* | *Pelargonic acid* |
| 133.0608 | 8.419 | Gly-Gly | *159.0663* | *13.607* | *Pimelic acid* |
| 133.0608 | 10.462 | Asn | *161.0455* | *15.26* | *3-Hydroxy-3-methylglutaric acid* |
| 136.0427 | 10.488 | Homocysteine | *165.0557* | *8.483* | *3-(2-Hydroxyphenyl)propionic acid or Tropic acid or 3-(4-Hydroxyphenyl)propionic acid* |
| 137.0445 | 11.049 | Hypoxanthine | *166.0146* | *15.745* | *Quinolinic acid* |
| 137.0709 | 7.355 | 1-Methylnicotinamide | *167.0211* | *8.944* | *Uric acid* |
| 138.055 | 10.447 | Anthranilic acid or Trigonelline | *167.9972* | *11.177* | *Cysteic acid* |
| 138.0913 | 8.385 | Tyramine | *171.1391* | *7.965* | *Decanoic acid* |
| 139.0502 | 8.283 | Urocanic acid | *172.0979* | *8.082* | *N-Acetylleucine* |
| 146.0924 | 8.28 | 4-Guanidinobutyric acid | *173.0455* | *8.223* | *Shikimic acid* |
| 146.1176 | 7.613 | Acetylcholine | *174.0408* | *14.371* | *N-Acetylaspartic acid* |
| 147.0917 | 8.086 | 5£¬6-Dimethylbenzimidazole | *174.0561* | *8.463* | *Indole-3-acetic acid* |
| 148.0604 | 10.91 | Glu | *175.0248* | *8.43* | *Ascorbic acid* |
| 148.0604 | 12.111 | threo-beta-Methylaspartic acid | *175.036* | *15.246* | *N-Carbamoylaspartic acid* |
| 148.0604 | 12.644 | O-Acetylserine | *175.0473* | *8.828* | *Allantoic acid* |
| 150.0774 | 7.937 | 3-Methyladenine | *176.0387* | *8.669* | *N-Formylmethionine* |
| 152.0706 | 11.423 | N-Methylanthranilic acid | *178.051* | *8.544* | *Hippuric acid* |
| 153.0229 | 15.003 | 6-Mercaptopurine | *184.0017* | *12.321* | *O-Phosphoserine* |
| 154.0863 | 8.621 | Dopamine | *187.134* | *7.646* | *10-Hydroxydecanoic acid* |
| 157.076 | 21.649 | 3-Indoleacetonitrile | *188.0353* | *8.488* | *Kynurenic acid* |
| 159.0513 | 21.456 | Allantoin or Allantoin | *188.0564* | *13.335* | *N-Acetylglutamic acid* |
| 161.0921 | 9.236 | Ala-Ala | *190.0543* | *8.11* | *N-Acetylmethionine* |
| 161.1073 | 8.209 | Tryptamine | *191.0561* | *8.107* | *Quinic acid* |
| 162.0761 | 10.79 | 2-Aminoadipic acid | *193.0354* | *8.062* | *Galacturonic acid or Glucuronic acid* |
| 162.1125 | 8.508 | Carnitine | *199.1704* | *7.623* | *Lauric acid* |
| 163.1077 | 6.987 | 5-Hydroxylysine | *205.0362* | *8.356* | *6£¬8-Thioctic acid* |
| 163.1077 | 8.397 | 2-Deoxystreptamine | *209.0303* | *14.116* | *Mucic acid* |
| 163.123 | 5.283 | Nicotine | *209.0303* | *14.414* | *Glucaric acid* |
| 166.0532 | 11.608 | Methionine sulfoxide | *210.0285* | *12.364* | *Phosphocreatine* |
| 166.0723 | 8.241 | 3-Methylguanine or 7-Methylguanine or 3-Methylguanine or 7-Methylguanine | *213.017* | *11.018* | *2-Deoxyribose 1-phosphate* |
| 168.0655 | 8.678 | Pyridoxal | *217.986* | *12.599* | *p-Nitrophenyl phosphate* |
| 170.0812 | 9.063 | Noradrenaline | *218.067* | *12.119* | *O-Succinylhomoserine* |
| 170.0924 | 7.487 | 3-Methylhistidine | *218.1034* | *7.66* | *Pantothenic acid* |
| 175.0866 | 21.476 | Indole-3-acetamide | *229.0119* | *11.014* | *Ribose 1-phosphate or Ribulose 5-phosphate or Xylulose 5-phosphate* |
| 175.1077 | 9.427 | N-Acetylornithine | *229.1445* | *11.014* | *Dodecanedioic acid* |
| 175.1077 | 11.372 | N5-Ethylglutamine | *231.0775* | *7.717* | *Nalidixic acid* |
| 176.0666 | 10.11 | Guanidinosuccinic acid | *243.0809* | *7.63* | *Biotin* |
| 179.0485 | 9.261 | Cys-Gly | *253.087* | *7.69* | *Ketoprofen* |
| 180.0867 | 9.341 | Glucosamine or Galactosamine or Mannosamine | *258.0384* | *8.882* | *Glucosamine 6-phosphate* |
| 181.0641 | 8.513 | Methionine sulfoximine | *261.0381* | *9.897* | *Sorbitol 6-phosphate* |
| 181.076 | 8.081 | o-Phenanthroline | *264.952* | *18.376* | *2£¬3-Diphosphoglyceric acid* |
| 182.0812 | 11.336 | Tyr | *283.0684* | *7.5* | *Xanthosine* |
| 184.0733 | 20.154 | Phosphorylcholine | *294.0094* | *7.667* | *Diclofenac* |
| 188.1757 | 6.44 | N8-Acetylspermidine | *300.049* | *9.163* | *N-Acetylglucosamine 6-phosphate* |
| 189.1234 | 9.72 | Gly-Leu | *300.049* | *9.613* | *N-Acetylglucosamine 1-phosphate* |
| 189.1234 | 11.417 | N6-Acetyllysine | *304.034* | *7.596* | *cCMP or 2'£¬3'-cCMP* |
| 189.1346 | 7.204 | Homoarginine | *306.0497* | *9.74* | *dCMP* |
| 189.1598 | 7.237 | N6,N6,N6-Trimethyllysine | *307.0337* | *9.951* | *dUMP* |
| 191.1026 | 9.05 | 2,6-Diaminopimelic acid | *308.0987* | *7.188* | *N-Acetylneuraminic acid* |
| 196.0968 | 9.453 | Tyrosine methyl ester | *308.9782* | *16.014* | *Ribulose 1£¬5-diphosphate* |
| 198.0761 | 11.473 | DOPA | *312.0503* | *7.483* | *3'£¬5'-Cyclic dAMP* |
| 198.0873 | 9.84 | N-Acetylhistidine | *322.0446* | *9.764* | *2'-CMP or CMP* |
| 204.1131 | 9.032 | Tryptophanamide | *330.0609* | *9.409* | *dAMP* |
| 204.123 | 8.876 | O-Acetylcarnitine | *337.0555* | *9.483* | *5-Aminoimidazole-4-carboxamide ribotide* |
| 206.0668 | 21.412 | Lipoamide | *346.0558* | *9.32* | *AMP or dGMP* |
| 209.0921 | 9.878 | Kynurenine | *346.0558* | *9.778* | *3'-AMP* |
| 221.1132 | 9.715 | N-Acetylglucosylamine | *351.2177* | *6.851* | *Prostaglandin E2* |
| 222.0972 | 21.531 | N-Acetylglucosamine | *353.2333* | *6.846* | *Prostaglandin F2alpha* |
| 223.0747 | 9.899 | Cystathionine | *363.0347* | *12.614* | *XMP* |
| 225.087 | 9.748 | 3-Hydroxykynurenine | *369.0675* | *9.567* | *Digalacturonic acid* |
| 228.0979 | 9.457 | 2'-Deoxycytidine | *371.5383* | *11.183* | *NADPH_divalent* |
| 233.1285 | 21.444 | Melatonin | *391.5713* | *7.791* | *FAD_divalent* |
| 241.0311 | 10.976 | Cystine | *417.0169* | *10.785* | *P1£¬ P4-Di(adenosine-5') tetraphosphate_divalent* |
| 241.1295 | 6.911 | Homocarnosine or Anserine | *417.5713* | *10.024* | *Isobutyryl CoA_divalent* |
| 245.2336 | 5.621 | N1-Acetylspermine | *421.0753* | *8.257* | *Trehalose 6-phosphate* |
| 247.1401 | 10.597 | Octopine | *426.0221* | *10.802* | *ADP or dGDP* |
| 249.0635 | 10.461 | Pyridoxamine 5'-phosphate | *427.0062* | *10.965* | *IDP* |
| 251.0696 | 12.75 | gamma-Glu-Cys | *462.0668* | *13.818* | *Adenylosuccinic acid* |
| 252.1091 | 9.664 | 2'-Deoxyadenosine | *487.1001* | *6.741* | *CDP-choline* |
| 256.104 | 10.923 | 7£¬8-Dihydroneopterin | *490.9776* | *12.05* | *dITP* |
| 258.1101 | 21.003 | Glycerophosphocholine | *505.9885* | *11.792* | *ATP* |
| 265.1123 | 6.655 | Thiamine | *506.9725* | *11.841* | *ITP* |
| 268.104 | 9.856 | Adenosine | *558.0644* | *8.418* | *ADP-ribose* |
| 268.104 | 11.445 | 2'-Deoxyguanosine | *563.0685* | *8.433* | *dTDP-glucose* |
| 277.103 | 10.921 | Glu-Glu | *565.0478* | *8.635* | *UDP-glucose or UDP-galactose* |
| 282.1197 | 9.914 | 1-Methyladenosine | *579.027* | *11.019* | *UDP-glucuronic acid* |
| 291.1299 | 9.47 | Argininosuccinic acid | *588.075* | *8.237* | *ADP-glucose or GDP-fucose* |
| 297.0582 | 21.35 | Disulfiram | *604.0699* | *8.198* | *GDP-mannose or GDP-galactose* |
| 298.0968 | 9.893 | 5'-Deoxy-5'-methylthioadenosine | *606.0743* | *8.467* | *UDP-N-acetylglucosamine* |
| 307.1765 | 10.624 | Nalpha-Benzenol arginine ethylester | *613.14* | *8.087* | *CMP-N-acetylneuraminate* |
| 307.9778 | 12.157 | 3-Iodotyrosine | *663.0859* | *6.504* | *Nicotinamide hypoxanthine dinucleotide* |
| 332.07 | 13.182 | Piroxicam | *663.0859* | *8.252* | *Deamido-NAD+* |
| 377.1456 | 21.504 | Riboflavin | *664.1175* | *8.215* | *NADH* |
| 380.1122 | 13.656 | S-Lactoylglutathione | *686.1416* | *8.008* | *3'-Dephospho CoA* |
| 385.1289 | 8.724 | S-Adenosylhomocysteine |  |  |  |
